# Response and Toxicity of Repeated Isolated Limb Perfusion (re-ILP) for Patients With In-Transit Metastases of Malignant Melanoma

**DOI:** 10.1245/s10434-018-07143-4

**Published:** 2019-01-07

**Authors:** Valerio Belgrano, Jessica Pettersson, Jonas A. Nilsson, Jan Mattsson, Dimitrios Katsarelias, Roger Olofsson Bagge

**Affiliations:** 1 Department of Surgery, Institute of Clinical Sciences, Sahlgrenska Academy at the University of Gothenburg, Sahlgrenska University Hospital, 413 45 Gothenburg, Sweden; 2 Sahlgrenska Cancer Center, University of Gothenburg, 405 30 Gothenburg, Sweden

## Abstract

**Background:**

Isolated limb perfusion (ILP) is a safe and well-established treatment for in-transit metastases of melanoma. In case of relapse or disease progression, ILP can be repeated (re-ILP). This study aimed retrospectively to analyze a large consecutive series of re-ILP and compare clinical outcomes with first-time ILP.

**Method:**

Between 2001 and 2015, 290 consecutive patients underwent 380 ILPs. Of these, 90 were re-ILPs including 68 second ILPs, 16 third ILPs, 4 fourth ILPs, and two fifth ILPs. The study evaluated response (using World Health Organization [WHO] criteria), local toxicity (using the Wieberdink scale), and complications (using Clavien–Dindo).

**Results:**

The results were compared between the first ILP, the second ILP, and the third to fifth ILP. The overall response rate was respectively 83%, 80% and 68%, with a complete response (CR) rate of 60%, 41%, and 59%. In the re-ILP group, the patients with a CR after the first ILP had a 65% CR rate after the second ILP compared with 8% for the patients without a CR (*p *= 0.001). The risk for local toxicity or complications was not increased after re-ILP. The median overall survival periods were respectively 34, 41, and 93 months (*p *= 0.02).

**Conclusion:**

As a therapeutic option, ILP can be repeated safely for in-transit metastases of melanoma, achieving similar high response rates without increasing complications or toxicity. Re-ILP is mainly indicated for patients who already had a CR after the first ILP, whereas other treatment options should be considered for primary non-responders.

**Electronic supplementary material:**

The online version of this article (10.1245/s10434-018-07143-4) contains supplementary material, which is available to authorized users.

Malignant melanoma accounts for the vast majority of all deaths due to skin cancer and is the fifth most common malignancy, with a progressively increasing incidence.^1^,^2^ For the majority of patients, malignant melanoma is diagnosed at an early stage and has a favorable prognosis, but for about 5–8% of patients with recurrences of melanoma, metastases will develop within the intradermal and subcutaneous lymphatic vessels, known as in-transit metastatic disease.^3^,^4^

Simple local surgical resection is the primary treatment option when possible. However, for patients with a high tumor burden or rapidly recurrent disease in the extremities, isolated limb perfusion (ILP) or isolated limb infusion (ILI) are regional treatment options to be considered. Both methods also can be used to gain locoregional control of extremity sarcomas, and for more rare conditions such as Merkel cell carcinoma (MCC), squamous cell carcinoma (SCC), and cutaneous lymphomas.^5^,^6^ Other, rapidly expanding local treatment options are electrochemotherapy (ECT),^7^ talimogene laherparepvec (T-VEC),^8^ and Rose Bengal.^9^

The ILP method, initially described by Creech et al.,^10^ is performed by open surgical access to the central venous and arterial blood flow of the limb, which is proximally isolated by a tourniquet and then connected to an extracorporeal oxygenator. A high concentration of a chemotherapeutic drug is then circulated through the targeted limb only. Continuous leakage monitoring and adjustments of flow are performed throughout the procedure to minimize potential systemic side effects of the chemotherapeutic agent.

The ILP approach has proved to be effective and safe with a high overall response rate (ORR), up to 90%, and low rates of regional and systemic toxicity.^11^ For patients not responding completely or having a recurrence on the same limb after a previous ILP, a repeated procedure (re-ILP) is possible.^12^–^14^ This report aims to describe our experience with re-ILP for melanoma patients in terms of response rate, toxicity, and survival, and to compare these findings with outcomes for first-time ILP.

## Methods

### Patients

During a 15-year period between January 2001 and December 2015, 290 patients with in-transit metastasis of melanoma underwent a total of 380 ILPs at our institution. Of these patients, 68 underwent a second ILP, 16 had a third ILP, 4 had a fourth ILP, and 2 had a fifth ILP, for a total of 90 re-ILPs. The patient characteristics are summarized in Table 1. The study was approved by the Regional Ethical Review Board at the University of Gothenburg.

**Table 1:** Patient and tumor characteristics

	1st ILP (*n *= 290) *n* (%)	2nd ILP (*n *= 68) *n* (%)	3rd ILP (*n *= 16) *n* (%)	4th ILP (*n *= 4) *n* (%)	5th ILP (*n *= 2) *n* (%)	Total (*n *= 380) *n* (%)
*Gender*						
Female	163 (56)	44 (65)	12 (75)	2 (50)	1 (50)	222
Male	127 (43)	24 (35)	4 (25)	2 (50)	1 (50)	158
Median age: years (range)	68 (23–93)	69 (31–85)	70 (55–80)	62 (56–82)	63 (62–64)	68 (23–93)
*Stage*						
N2c	144 (50)	32 (47)	6 (38)	1 (25)	1 (50)	184
N3	128 (44)	31 (46)	9 (56)	3 (75)	1 (50)	172
M1	18 (6)	5 (7)	1 (6)	0 (0)	0 (0)	24
*Vascular access*						
Axillary/brachial	46 (16)	10 (15)	1 (6)	0 (0)	0 (0)	57
External iliac	84 (29)	8 (12)	1 (6)	0 (0)	1 (50)	94
Femoral	160 (55)	50 (73)	14 (88)	4 (100)	1 (50)	229
*Chemotherapy*						
Melphalan	263 (91)	14 (21)	1 (6)	1 (25)	1 (50)	280
TNF + melphalan	27 (9)	54 (79)	15 (94)	3 (75)	1 (50)	100
*Largest tumor diameter (mm)*						
≤ 30	136 (81)	32 (80)	10 (100)	2 (100)	2 (100)	181
> 30	31 (19)	8 (20)	0 (0)	0 (0)	0 (0)	39
*No. of tumors*						
≤ 10	147 (72)	37 (67)	7 (54)	2 (50)	0 (0)	193
> 10	57 (28)	18 (33)	6 (46)	2 (50)	2 (100)	85

*ILP* isolated limb perfusion, *TNF* tumor necrosis factor

### Isolated Limb Perfusion

The vascular system of the treated limb was isolated by cannulation and clamping of the major artery and vein, which then were connected to an oxygenated extracorporeal circuit. The remaining collateral vessels were compressed with a proximal inflatable tourniquet (Zimmer disposable tourniquet) or an Esmarch bandage.

For first-time ILPs, melphalan (M-ILP) (Alkeran; GlaxoSmithKline Pharmaceuticals, Research Triangle Park, NC, USA) was used. The addition of tumor necrosis factor-alpha (TNF-alpha, Beromun; Boehringer Ingelheim, Germany) (TM-ILP) was considered primarily for bulky melanomas (tumors with a diameter > 30 mm) and also for re-ILPs.

In M-ILP, the dose of melphalan was calculated according to limb volume using 13 mg/L for upper-limb perfusions and 10 mg/L for lower-limb perfusions. Before 2012, melphalan was administered as three bolus doses, with 50% of the total dose administered initially and the remaining 50% administered in two equivalent doses at 30-min intervals, with a total perfusion time of 90 min.

In 2012, the administration of melphalan was changed to a 20-min infusion into the perfusion circuit and a total perfusion time of 60 min.^15^ For patients receiving TM-ILP, a bolus dose of TNF-alpha was injected into the perfusion circuit (3 mg for the upper limb and 4 mg for the lower limb) provided the limb tissue temperature had reached 38 °C. Then, 30 min later, melphalan was administered during a 20-min infusion. The total perfusion time was 90 min. All treatments were performed with the patient under mild hyperthermia (39–40 °C). No adjustments in perfusion characteristics were made for re-ILPs.

During the procedure, continuous leakage monitoring was performed with a precordial scintillation probe (MedicView, Gothenburg, Sweden) to detect and measure leakage of technetium-99 m-labeled human serum albumin (Vasculosis; Cis Bio, Gif-sur-Yvette, France) injected into the perfusion circuit. After perfusion, the limb was rinsed with at least 1–2 L (upper limb) or 3–4 L (lower limb) of Ringer’s solution (Ringer Acetate; Baxter, Stockholm, Sweden). For re-ILPs, the same surgical access through the scar of the previous incisions was used, and cannulation was feasible in all cases.

### Response

Response was evaluated at 3 months according to the World Health Organization (WHO) criteria.^16^ A complete response (CR) was defined as the disappearance of all lesions. Partial response (PR) was defined as more than a 50% decrease in the total tumor burden. Progressive disease (PD) was defined as an increase of more than 25% in existing lesions or the appearance of new lesions. Stable disease (SD) was defined as a result that failed to meet the criteria for CR, PR or PD.

### Toxicity and Complications

Local toxicity was monitored and evaluated by a physician up to 3 months after ILP and graded as the worst toxicity during that time according to the Wieberdink classification as follows: grade 1 (no reaction), grade 2 (erythema or swelling), grade 3 (major erythema, swelling, or blistering), grade 4 (extensive epidermolysis and/or damage to deep tissues, causing final functional disorders, risk, or manifest compartment syndrome), and grade 5 (reaction that could necessitate amputation).^17^ Complications within 30 days post-operatively were graded according to Clavien-Dindo.^18^

### Statistical Analysis

Statistical analysis was performed using IBM SPSS Statistics, version 25.0 (IBM Corp., Armonk, NY, USA). Survival estimates were analyzed using the Kaplan–Meier method and the log-rank test. Cox-regression analysis using the enter method was used to perform multivariate analysis for survival, measured from the time of the first ILP. Logistic regression using the enter method was used to analyze predictive factors for response and toxicity. A *p* value lower than 0.05 was regarded as significant.

## Results

### Time Between ILPs

The median time between ILPs was 13 months (range, 3–97 months) between the first and second ILPs, 12 months (range, 5–36 months) between the second and the third ILPs, 16 months (range, 5–24 months) between the third and fourth ILPs, and 10 months (range, 6–14 months) between the fourth and fifth ILPs. For the patients who had a CR after the first ILP, the median time to the second ILP was 15 months, compared with 10 months for the patients who had no CR after the first ILP (*p *= 0.01).

### Response

Response was evaluable for 298 ILPs (79%), and 57 ILPs were found to be adjuvant (no clinically evaluable tumor). Response data were missing or considered not reliable for 25 ILPs. The overall response rate was 83% after the first ILP, 80% after the second ILP, and 68% after the third to fifth ILPs (*p* = 0.27). The CR rates were respectively 60%, 41%, and 59% (*p* = 0.04).

**Fig. 1:**
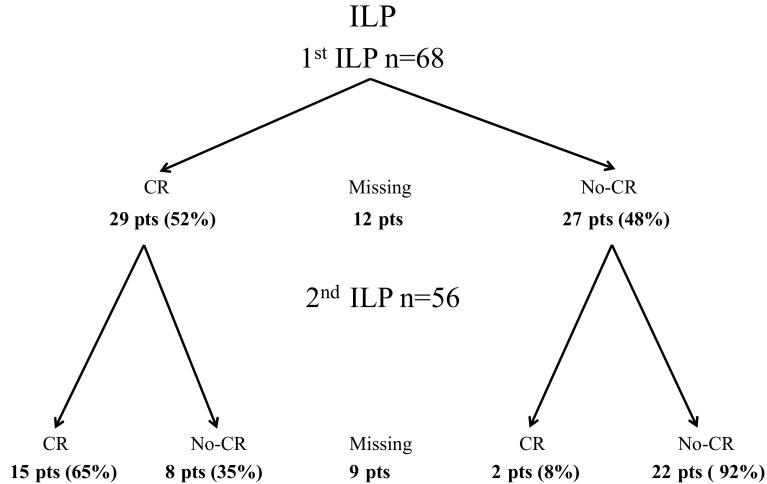
Diagram of repeated isolated limb perfusion (ILP) (re-ILP) divided by complete response (CR) or no CR

In the re-ILP group, 29 patients showed a CR after the first ILP (52%), and the CR rate after their second ILP was 65%. Of the 57 patients, 27 (48%) showed no CR after their first ILP, and the CR rate after their second ILP was 8% (Fig. 1) (*p* = 0.001).

Comparison of the evaluable response rates after the first ILP showed no difference between the patients who underwent only one ILP (*n* = 164) and those who later underwent a re-ILP (*n* = 57). The overall response rate at their first ILP was 84% versus 79% (*p* = 0.43), and the CR rate was 62% versus 53% (*p* = 0.21).

The analysis of all the evaluable ILPs in a multivariate logistic regression for CR showed that the only independent predictive factor was the number of tumors (≤ 10 vs. > 10; odds ratio [OR], 0.3; *p *< 0.001) (Table 2).

**Table 2:** Uni- and multivariate logistic regression for complete response

	Univariate analysis			Multivariate analysis		
	OR	95 % CI	*p* Value	OR	95 % CI	*p* Value
Age (years)	1.0	0.9–1.0	0.2	1.0	0.9–1.1	0.07
*Gender*						
Female^a^ versus male	0.9	0.6–1.4	0.6	0.9	0.5–1.8	0.9
*Stage*						
N2c^a^ versus N3	0.8	0.5–1.3	0.4	1.6	0.8–3.0	0.1
N2c^a^ versus M1	0.3	0.1–0.8	0.02	0.4	0.1–1.5	0.2
*No. of tumors*						
≤ 10^a^ versus > 10	0.2	0.1–0.4	< 0.001	0.3	0.1–0.5	< 0.001
*Tumor size*						
≤ 30^a^ versus > 30 mm	0.5	0.2–1.0	0.05	0.6	0.2–1.4	0.3
*Chemotherapy*						
M-ILP^a^ versus TM-ILP	0.5	0.3–0.8	0.005	0.5	0.2–1.4	0.2
*No. of perfusions*						
1st ILP versus 2nd ILP	0.5	0.3–0.9	0.1	0.8	0.3–2.5	0.7
1st ILP versus 3rd–5th ILP	1.0	0.4–2.4	0.9	4.6	0.9–24.2	0.07

*OR* odds ratio, *CI* confidence interval, *ILP* isolated limb perfusion, *M-ILP* melphalan based isolated limb perfusion, *TM-ILP* TNF-alpha and melphalan based isolated limb perfusion

^a^Reference category

### Melphalan and TNF-Alpha

Among the first ILPs (*n* = 290), 263 were M-ILPs (91%) and 27 were TM-ILPs (9%). The main indication for TM-ILP was bulky disease (Table 1). The response was evaluable for 76% of the patients with an ORR of 85% for M-ILP and 67% for TM-ILP (*p* = 0.045). The CR rate was 63% in the M-ILP group and 33% in the TM-ILP group (*p* = 0.007).

Among the 90 re-ILPs performed, 19% were M-ILPs and 81% were TM-ILPs (Table 1). An M-ILP was performed instead of a TM-ILP due to leakage problems (*n* = 9), technical problems with reperfusion (*n* = 2), or advanced comorbidity or age (*n* = 4), and one re-ILP was adjuvant after a CR with a surgically removed recurrence. In the reperfusion group, the ORR was 55% for M-ILP and 81% for TM-ILP (*p* = 0.13). The CR rate was 36% for M-ILP and 47% for TM-ILP (*p* = 0.77).

### Toxicity and Complications

Local toxicity was evaluable for 81% of all ILPs, and 63% were grade 1 or 2, 30% were grade 3, and 7% were grade 4. One patient experienced a grade 5 toxicity necessitating an amputation (Table 3). The multivariate analysis showed no predictive factor for low versus high toxicity (grade 1 or 2 vs. grades 3–5) (Table S1).

**Table 3:** Outcome: response rates and local toxicity

	1st ILP *n* (%)	2nd ILP *n* (%)	3rd ILP *n* (%)	4th ILP *n* (%)	5th ILP *n* (%)	Total *n* (%)
*Response (WHO)*						
CR	131 (60)	23 (41)	10 (63)	3 (75)	0 (0)	167 (56)
PR	51 (23)	22 (39)	2 (12)	0 (0)	0 (0)	75 (25)
SD	24 (11)	8 (14)	3 (19)	1 (25)	1 (50)	37 (12)
PD	14 (6)	3 (5)	1 (6)	0 (0)	1 (50)	19 (7)
*Toxicity (Wieberdink)* ^*a*^						
1	14 (6)	2 (4)	0 (0)	0 (0)	0 (0)	16 (5)
2	141 (57)	28 (62)	7 (58)	3 (75)	0 (0)	179 (58)
3	74 (30)	11 (24)	5 (42)	1 (25)	1 (100)	92 (30)
4	17 (7)	4 (9)	0 (0)	0 (0)	0 (0)	21 (7)
5	1 (0.5)	0 (0)	0 (0)	0 (0)	0 (0)	1 (0.3)
*Complications (Clavien–Dindo)* ^*b*^						
1	1 (1.5)	0 (0)	0 (0)	0 (0)	0 (0)	1 (0.6)
2	5 (7)	1 (1.5)	2 (13)	1 (25)	0 (0)	9 (6)
3	1 (1.5)	2 (3)	0 (0)	0 (0)	0 (0)	3 (2)
4	0 (0)	1 (1.5)	0 (0)	0 (0)	0 (0)	1 (0.5)
5	0 (0)	1 (1.5)	0 (0)	0 (0)	0 (0)	1 (0.5)

*WHO* World Health Organization, *ILP* isolated limb perfusion, *CR* complete response, *PR* partial response, *SD* stable disease, *PD* progressive disease

^a^Toxicity is graded according to the Wieberdink scale

^b^Complications are graded according to Clavien–Dindo

Considering only patients who underwent a re-ILP, the toxicity after the first ILP was 70% grade 1 or 2, 27% grade 3, and 3% grade 4. For the second ILP, the toxicities were respectively 67%, 24%, and 9%, and for the third to fifth ILPs, the toxicities were respectively 59%, 41% and 0% (*p* = 0.664).

Complications within 30 days postoperatively were graded according to Clavien–Dindo. The first ILP resulted in 90% grade 0, 1.5% grade 1, 7.4% grade 2, 1.5% grade 3, and 0% grade 4 or 5 complications.

Re-ILP resulted in 89% grade 0, 0% grade 1, 4% grade 2, 2% grade 3, and 1% grade 4 complications. The complications did not differ between the first-time ILPs and the re-ILPs (*p* = 0.54).

### Survival

**Fig. 2:**
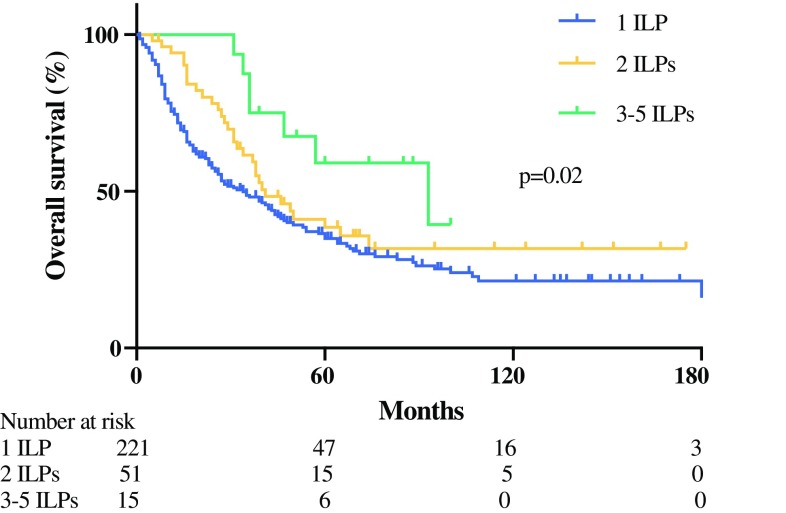
Overall survival. Median overall survival differed significantly depending on whether the patients were treated with one isolated limb perfusion (ILP) (34 months), two ILPs (41 months), or three to five ILPs (93 months) (*p *= 0.02)

The median overall survival (OS) was 39 months, with a 5-year survival of 37%. The median OS was 34 months for the patients treated with one ILP, 41 months for patients treated with two ILPs, and 93 months for those who underwent three to five ILPs (*p* = 0.02) (Fig. 2). In the multivariate analysis, the factors predictive for increased survival were lower stage, increased number of ILPs, and CR. When response from the multivariate analysis was excluded, stage and number of ILPs remained significant, but in this case also, the number of metastases was statistically significant (Table S2).

## Discussion

This study was a retrospective analysis of a prospective database at a single institution, with all the associated limitations. However, it is the largest series of re-ILPs reported to date and includes a consecutive series of all the patients treated in Sweden between 2001 and 2015.

In this study, the overall response rate for first-time ILPs was 85% (including a CR rate of 62%), which is comparable with previous results from our own institution.^15^,^19^ and also with other previous reports.^11^

On the contrary, when only the re-ILPs were analyzed, we found that our response rates were in the lower range, with an ORR of 77% and a CR rate of 52% compared with other major studies of re-ILP reporting an ORR of 71–96% and a CR rate of 62–76% (Table 4). 12–14 This result likely was due to a selection bias, with other institutions mainly performing re-ILPs for patients with recurrences after a complete or near-complete response following the previous ILP.^12^–^14^

**Table 4:** Overview of major studies reporting on repeat isolated limb perfusion (ILP) for in-transit melanoma metastases

Author	No. of re-ILPs	No. of patients	Toxicity 1–2 (Wieberdink) (%)	Complete response rate (%)
Grünhagen et al.^13^	25	21	72	76
Noorda et al.^14^	21	21	66	62
Deroose et al.^12^	37	32	70	66
Sahlgrenska University Hospital	90	68	63	52

*ILP* isolated limb perfusion

In our series, many patients who did not respond after the first ILP were treated with a re-ILP. However, the current results show that if the patient did not respond the first time, the probability for a response after a re-ILP was also low. In the modern treatment landscape these patients should be discussed primarily for other treatments and not be recommended for an additional ILP. However, for patients achieving a CR after the first ILP, re-ILP remains a treatment option with high response rates and no increased risk for toxicity or complications.

Of the included ILPs, 57 were adjuvant ILPs after resection of recurrent in-transit metastases. For these patients, the response rate cannot be reported, but they still were included in the analyses because they provided information about toxicity and also completed the consecutive series. Adjuvant perfusions are no longer performed at our institution, but in the first part of the study period, no other adjuvant therapy was available, and data from a randomized trial showed an increase in disease-free survival but without survival benefit.^20^

Two thirds of the patients experienced a grade 1 or 2 toxicity independently of the number of ILPs. These findings are similar to those of other series reporting grades 1 and 2 reactions of 66–73%.^11^–^14^ Severe reactions such as grade 4 toxicity were 7% in this series, which is somewhat higher than other reports of 0–5%.^11^–^14^ One reason could have been the long-term follow-up period for the patients, in which any deficit in limb sensitivity or motor function was regarded as a persistent grade 4 toxicity.

However, the most significant result was that the number of ILPs did not increase the risk for toxicity or complications. In our practice, we almost always use the previous incision also for a re-ILP. This makes the surgical access a little more challenging, but we have not experienced any problems with this approach. This also is supported by the complication data, which show no increase in complications in the re-ILP group compared with the first-time ILP group.

The use of TNF-alpha can be highly questioned, but our practice has been to consider adding TNF-alpha for bulky tumors (> 30 mm) for first-time ILP and for all re-ILPs. In the group of first-time ILPs, the CR rate was 63% after M-ILP and 33% after TM-ILP. This difference was most probably due to the use of TNF-alpha only for bulky tumors, which is a known negative predictive factor for response. In the re-ILPs, TNF-alpha was not used for 17 patients, and in this group, both the OR and CR rates were lower but not statistically significant in either the uni- or multivariate analyses. The well-known randomized trial of Cornett et al.^21^ was not able to show any difference in response with the addition of TNF-alpha, but many European institutions are using the same treatment schedule as we use.^22^

No conclusions about TNF-alpha in the re-ILP setting can be drawn based on the current results, and the exact role of TNF-alpha remains to be defined. At our institution, the rationale for adding TNF-alpha in the re-ILP setting has mainly been based on those patients who did not respond after the first ILP, for whom the treatment needed modification to increase the efficiency. Based on the current data, we no longer recommend re-ILP for patients who do not respond the first time, and thus indirectly, the use of TNF-alpha also can be questioned.

The 5-year OS after the first ILP was 36%, almost identical to the results shown by Moreno-Ramirez et al.,^11^ emphasizing that patients with in-transit metastases are a high-risk group, with adjuvant therapies likely having a crucial role in improving survival. The OS was significantly higher for the patients receiving more ILPs, but this most probably was due to a selection bias and not an effect of the ILP itself. Patients with a longer survival have a higher risk of locoregional relapse. Therefore, they are more likely to be considered for a re-ILP. Previous reports show similar findings, with increased survival for the re-ILP groups,^14^–^16^ but any direct comparison of survival times is not possible because both the indications and the case mix are different. However, hypothetically, an improved survival in the re-ILP group could have been related to a repeated immunologic effect, with some emerging data showing a correlation between immunologic factors and response.^23^,^24^ However, these data must be seen as speculative and do not justify any conclusion to date.

In the case of relapse or progression after a previous ILP, other options include ILI, which is a safe and easily repeatable technique, but with lower response rates and no difference in survival.^25^ In a smaller series of 44 patients undergoing either repeated ILP or ILI, no difference was observed in time to local recurrence or toxicities.^26^ Potentially, ILI could be easier to perform from a technical point of view, but in our practice, we also add TNF-alpha in the re-ILP setting, and this option is then not available in ILI.^25^ Other options include local injection therapies such as talimogene laherparepvec (TVEC),^27^ which is a genetically modified, live, attenuated herpes simplex virus type 1 designed to replicate in tumor cells and promote an enhanced antitumor response. In a randomized phase 3 trial, TVEC presented an ORR of 26%, including a CR of 11%.^28^

The development of new systemic drugs in recent years has radically changed melanoma treatment. The introduction of BRAF/MEK inhibitors as well as checkpoint inhibitors with anti-CTLA-4 and anti-PD-1 antibodies has offered new hope for patients with stage 4 melanoma. However, the specific response rates with these drugs for in-transit metastases have not been reported to date, but the response rates for inoperable stage 3 and stage 4 patients are inferior to the results achieved by ILP or ILI, with CR rates of approximately 5–15%.^29^–^31^ An interesting concept is the combination of regional therapies using modern immunotherapy, with a recently presented trial combining ILI and ipilimumab and showing a positive synergistic effect.^32^

In conclusion, ILP and re-ILP give high response rates, and the toxicity and complication rates do not depend on the number of perfusions performed for each patient. We still consider ILP as the best treatment option for achieving local control of in-transit metastases confined to the extremities. For recurrent in-transit metastases, re-ILP still has a role, especially if the patient responded after the first treatment. Considering the rapid evolution of both systemic and various local treatments, the exact role of ILP and re-ILP might have to be redefined in the near future.

## Electronic supplementary material

Below is the link to the electronic supplementary material.

Supplementary material 1 (DOCX 17 kb)
