# Supplementary material for: Response and Toxicity of Repeated Isolated Limb Perfusion (re-ILP) for Patients With In-Transit Metastases of Malignant Melanoma
Source: Ann Surg Oncol. 2019 Jan 7;26(4):1055–62. doi: 10.1245/s10434-018-07143-4 (PMC6399180; doi:10.1245/s10434-018-07143-4)
Supplement: Supplementary file 1 — Supplementary material 1 (DOCX 17 kb) [file 10434_2018_7143_MOESM1_ESM.docx]

Supplementary Table 1. Predictive factors for local toxicity (grade I-II vs. III-V).

|  | *Univariate analysis* | | | *Multivariate analysis* | | |
| --- | --- | --- | --- | --- | --- | --- |
|  | OR | 95% CI | p-value | OR | 95% CI | p-value |
| Age (per year) | 1.0 | 0.9-1.0 | 0.6 | 1.0 | 0.9-1.0 | 0.9 |
| Gender  Female* vs. Male | 0.8 | 0.5-1.3 | 0.3 | 1.1 | 0.6-2.0 | 0.8 |
| Stage  N2c* vs. N3  N2c* vs. M1 | 0.6  1.4 | 0.3-1.0  0.5-3.9 | 0.5  0.5 | 0.8  2.3 | 0.4-1.6  0.5-9.5 | 0.6  0.3 |
| Numbers of tumours  <=10* vs. >10 | 1.3 | 0.7-2.2 | 0.4 | 1.2 | 0.6-2.4 | 0.6 |
| Tumour size  <=30* vs. >30 | 1.4 | 0.6-3.2 | 0.4 | 1.6 | 0.6-4.4 | 0.3 |
| Chemotherapy  M-ILP* vs. TM-ILP | 1.0 | 0.6-1.8 | 0.9 | 0.8 | 0.3-2.2 | 0.7 |
| Number of perfusions  1^st^ ILP vs. 2^nd^ ILP  1^st^ ILP vs. 3^rd^-5^th^ ILP | 0.8  1.2 | 0.4-1.6  0.4-3.2 | 0.6  0.7 | 0.9  2.0 | 0.3-2.6  0.4-9.2 | 0.8  0.4 |
| Perfusion vessel  Brachial vs. External Iliac  Brachial vs. Femoral | 1.3  2.1 | 0.6-3.0  1.0-4.3 | 0.5  **0.04** | 0.7  2.7 | 0.2-3.2  1.0-7.5 | 0.7  0.06 |

Supplementary Table 2. Prognostic factors for survival.

|  | *Univariate analysis* | | | *Multivariate analysis* | | |
| --- | --- | --- | --- | --- | --- | --- |
|  | HR | 95% CI | p-value | HR | 95% CI | p-value |
| Age (per year) | 1.0 | 0.9 | 0.1 | 1.0 | 0.9-1.0 | 0.14 |
| Sex  Female* vs. Male | 1.5 | 1.1-2.0 | **0.005** | 1.1 | 0.7-1.7 | 0.6 |
| Stage  N2c* vs. N3  N2c* vs. M1 | 1.8  3.2 | 1.4-2.5  1.8-5.7 | **<0.001**  **<0.001** | 2.0  2.1 | 1.2-3.3  0.9-4.7 | **0.004**  0.07 |
| Numbers of tumours  <=10* vs. >10 | 1.5 | 1.0-2.2 | **0.03** | 1.5 | 0.8-2.5 | 0.13 |
| Tumour size  <=30* vs. >30 | 2.2 | 1.4-3.5 | **0.001** | 1.9 | 0.9-3.6 | 0.5 |
| Chemotherapy  M-ILP* vs. TM-ILP | 2.3 | 1.5-3.5 | **<0.001** | 1.1 | 0.5-2.4 | 0.7 |
| Number of perfusions  1 ILP vs. 2 ILPs  1 ILP vs. 3-5 ILPs | 0.7  0.4 | 0.5-1.1  0.2-0.9 | 0.1  **0.03** | 0.6  0.07 | 0.3-1.1  0.01-0.5 | 0.1  **0.009** |
| Response at 1^st^ ILP  CR vs. PR  CR vs. SD  CR vs. PD | 2.3  3.6  4.6 | 1.4-3.8  2.0-6.5  2.2-9.7 | **0.01**  **<0.001**  **<0.001** | 1.7  3.5  3.3 | 0.9-3.1  1.8-6.9  1.4-8.0 | 0.063  **<0.001**  **0.006** |
